# Mitochondrial dysfunction in urologic disease

**DOI:** 10.1038/s41391-026-01097-5

**Published:** 2026-03-24

**Authors:** Alexis E. Adrian, Natalie J. Boucher, Kevin T. McVary, Donald B. DeFranco, William A. Ricke

**Affiliations:** 1 Department of Urology, University of Wisconsin- Madison, Madison, WI USA; 2 Department of Human Physiology, University of Oregon, Eugene, OR USA; 3 Center for Male Health, Loyola University Chicago, Stritch School of Medicine, Maywood, IL USA; 4 Department of Pharmacology and Chemical Biology, University of Pittsburgh School of Medicine, Pittsburgh, PA USA

**Keywords:** Urogenital diseases, Prostatic diseases, Medical research

## Abstract

**Purpose:**

Lower urinary tract symptoms (LUTS) can result from several conditions, including but not limited to benign prostatic hyperplasia, diabetes, stress urinary incontinence, and metabolic syndrome. In 2011, it was estimated that nearly 2 billion people (45.2% of the world population) experience one or more LUTS. To date, there is no single underlying mechanism identified in lower urinary tract symptoms or the associated conditions. However, a recent body of evidence has demonstrated that mitochondrial dysfunction may contribute to conditions associated with LUTS. Furthermore, patients with inherited mitochondrial disease exhibit lower urinary tract symptoms at rates above the general population. Given that mitochondrial dysfunction is an under-investigated area of urinary disease research, this review aims to summarize the existing literature in the field.

**Methods:**

PubMed was used as the primary database for literature search. Keywords included “lower urinary tract symptoms”, “mitochondria”, “urinary dysfunction”; additionally, once identified, further disease-specific searches were performed (e.g., “urinary dysfunction in type 1 diabetes”). Studies published earlier than August 2025 were included, and a specific focus was placed on clinical or human studies. Urologic conditions lacking at least three publications investigating mitochondrial dysfunction were not included; studies examining mitochondrial dysfunction in urologic cancers were also outside the scope of this review.

**Results and conclusions:**

Mitochondrial dysfunction is a potential novel intersection of diseases associated with LUTS, including aging, metabolic syndrome, diabetes, stress urinary incontinence, benign prostatic hyperplasia, and overactive bladder. While initial studies are promising, more investment into identifying underlying mechanisms and new potential treatments is needed. Additionally, as mitochondrial modulators undergo trial as treatments in other conditions, careful consideration should be given to their potential as treatments for LUTS.

**Figure:**
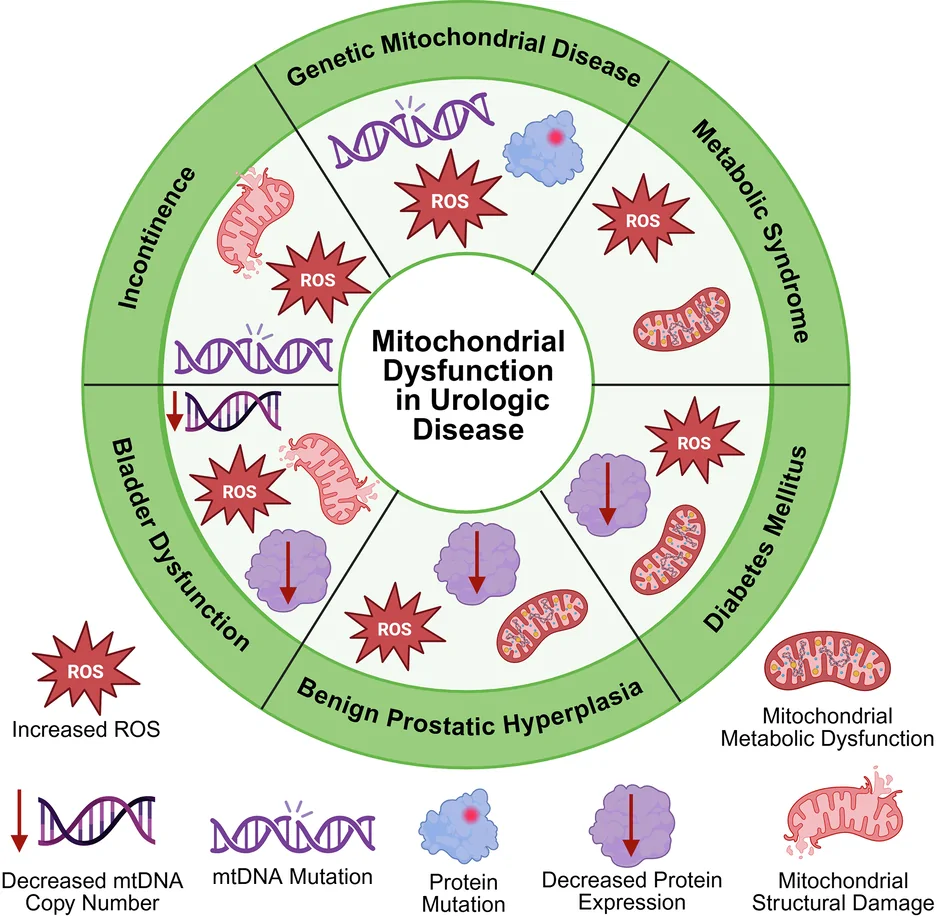

## Introduction

Mitochondria are powerful organelles that contribute to cellular metabolism, signal transduction, and cellular homeostasis. Mitochondrial dysfunction is a broad term used to define many cellular and molecular processes that have far-reaching implications in human disease, which is more broadly reviewed in other recent publications [1, 2]. Many fields have identified mitochondria as key contributors to disease pathology including but not limited to neurology, cardiology, gerontology, endocrinology, and others [3]. There is evidence, outlined herein, that mitochondrial dysfunction is present in urologic disease and related comorbidities of lower urinary tract symptoms (LUTS), but no underlying causal mechanism has been defined. It remains unknown what role mitochondrial dysfunction plays in many urologic conditions and whether targeting said dysfunction has therapeutic potential clinically. The purpose of this review is to first summarize the most fundamental parts of mitochondrial biology and second, review existing literature on the role of mitochondrial dysfunction in non-malignant urologic diseases.

## Mitochondrial genome and proteome

**Fig. 1:**
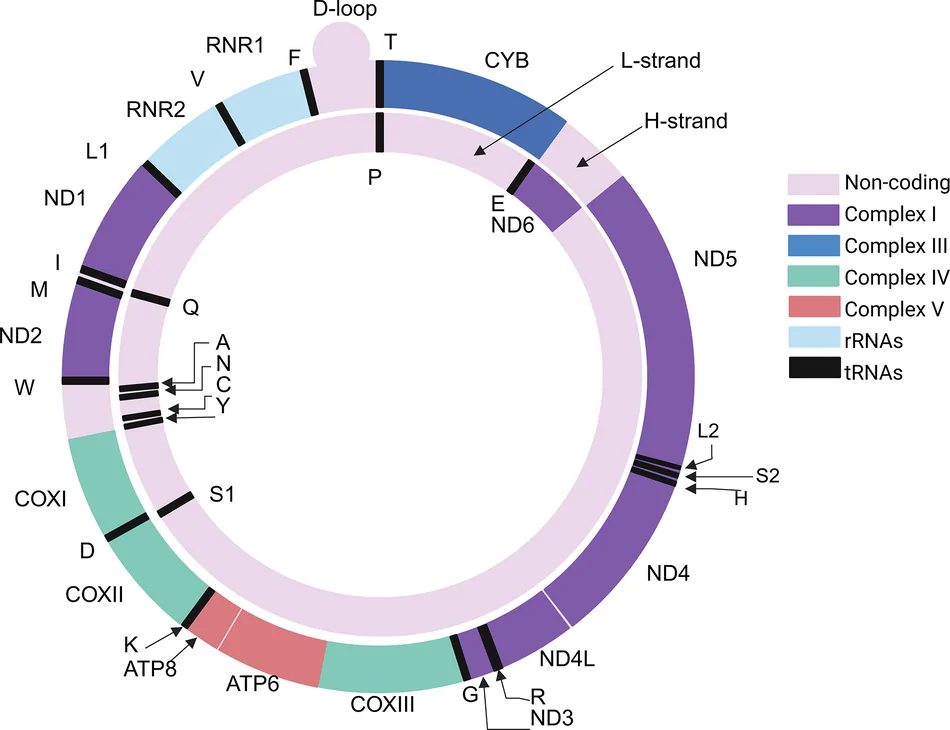
Map of the human mitochondrial genome. mtDNA is circular in structure and contains heavy (H) and light (L) strands. Coding and noncoding regions are color coded to their corresponding protein complex or RNA. Proteins are labeled to their corresponding gene region as are rRNA and tRNA.

Mitochondria are unique organelles, due to their origin from independent endosymbiotic bacteria. Each mitochondrion has a double membrane structure, with an outer membrane, intermembrane space, inner membrane, and matrix. Additionally, mitochondria exist in complex, dynamic, interconnected networks. Mitochondria are involved in many cellular processes, including energy homeostasis, redox environments, signaling through the generation of reactive oxygen species (ROS) and other small molecules, calcium flux, metabolite and protein transport, and are contributors to programmed cell death pathways. To some extent, these organelles behave as independent cells; each mitochondrial membrane encompasses respiratory complexes and transport proteins, mitochondrial DNA (mtDNA) with its own replication machinery, and the enzymes and macromolecular components required to perform transcription and translation (rRNA, tRNA, ribosomes, etc.) [4]. Mitochondrial DNA is circular and encodes 37 genes (Fig. 1) [5]. Of the 37 genes, 13 are polypeptides, all of which are subunits of respiratory complexes I, III, IV, and V of the electron transport chain (ETC) [6–8]. Unlike nuclear DNA, mtDNA is not protected by histones; therefore, it is more prone to chemical damage from endogenous and exogenous sources [9–12]. For example, its proximity to oxidative phosphorylation (OXPHOS) exposes it to ROS, which are produced during normal function of the ETC. Given the highly reactive nature of ROS, mtDNA is highly susceptible to oxidative damage [13–15]. Mitochondria have co-opted nuclear mechanisms for DNA damage repair; despite these adapted mechanisms, the mitochondrial genome is still prone to mutations, with a rate 10–20 times higher than the nuclear genome [12]. Each mitochondrion contains multiple copies of mtDNA. This distinct redundancy leads to a genomic phenomenon that only occurs in mitochondria and chloroplasts, known as heteroplasmy. Heteroplasmy is the presence of two or more genomic subpopulations within a given cell or tissue. While some degree of heteroplasmy occurs through inherited mutations or subpopulations that have developed, deleterious accumulation of heteroplasmy is associated with increased risk and incidence for diseases such as type 2 diabetes, cancer, and cardiovascular diseases [16–19]. Increased heteroplasmy also occurs with aging and may be related to late-onset diseases (e.g., idiopathic Parkinson’s disease) and increased frailty [17, 20]. Because mtDNA encodes exclusively for ETC polypeptides and transcription/translation machinery, heteroplasmy above a biochemical threshold negatively affects ETC function, thereby contributing to reduced metabolic homeostasis and elevated ROS.

In addition to mitochondrial encoded proteins, nuclear encoded proteins are trafficked into mitochondria and make up much of the mitochondrial proteome, including in the outer membrane and intermembrane space, as well as most inner membrane and matrix proteins [21]. In addition to nuclear-encoded proteins trafficked to the mitochondrion for bioenergetics, there are multiple nuclear-encoded regulators of other mitochondria processes, such as nuclear respiratory factors 1 and 2 (NRF-1/NRF-2), mitochondrial transcription factor A (TFAM), and peroxisome proliferator-activated receptor gamma coactivator 1α (PGC-1α), as well as many others [21]. Collectively, these proteins regulate expression and activity of the ETC proteins, stabilization and maintenance of mtDNA, and mitochondrial biogenesis. Much of mitochondrial regulatory function has a direct effect on metabolism, especially the ETC.

## Oxidative phosphorylation through the mitochondrial electron transport chain

**Fig. 2:**
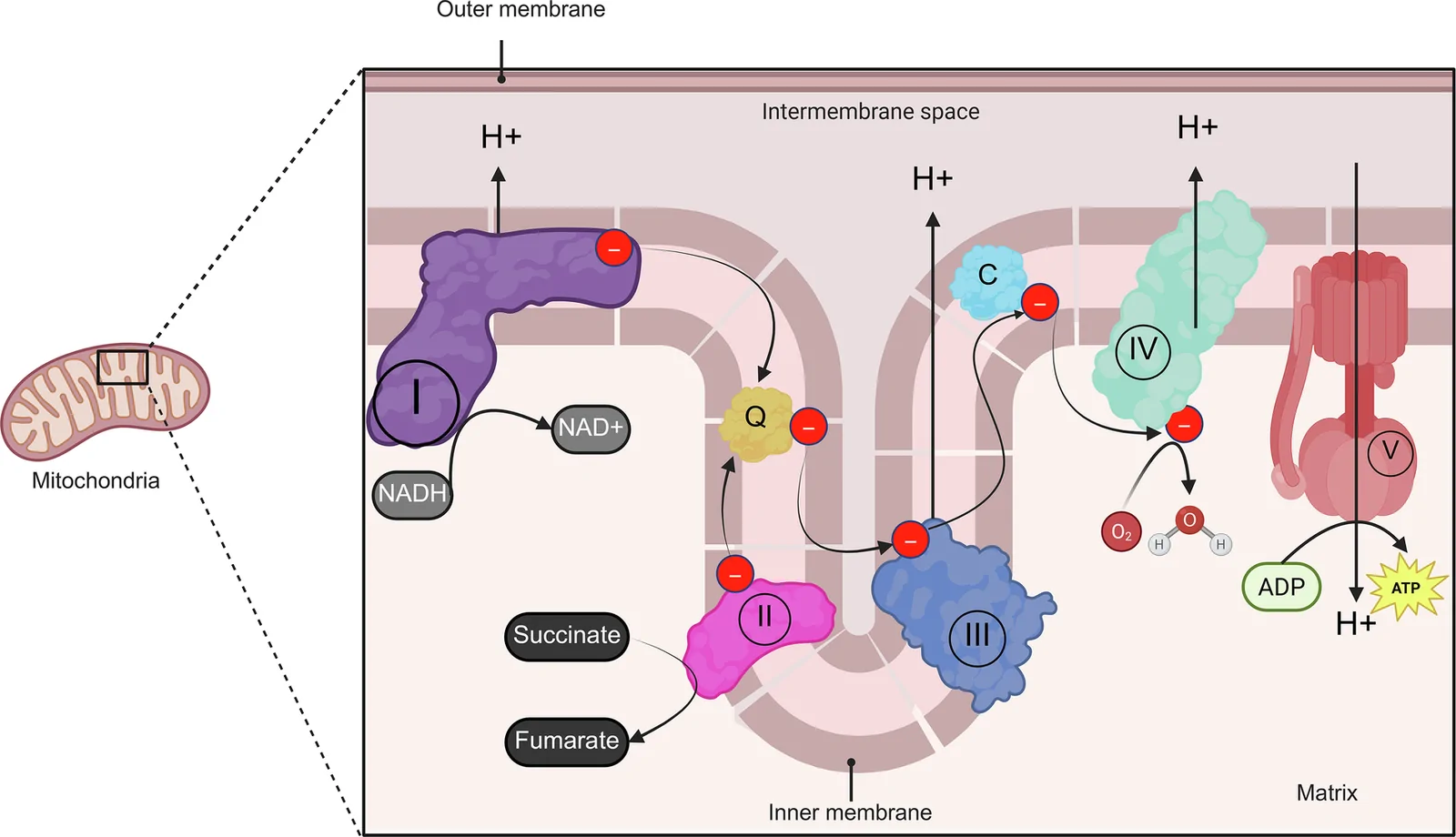
Diagram representation of the electron transport chain (ETC). Regions of the mitochondria are noted. Complexes I-V are labeled. Minus signs represent the flow of electrons. Q; coenzyme Q, C; cytochrome C.

Mitochondria generate the majority of adenosine triphosphate (ATP) in many cell types [22]. At the center of mitochondrial bioenergetics is OXPHOS, which is driven by the ETC (Fig. 2). The ETC is made up of five major protein complexes (I-V) and two carrier molecules, coenzyme Q and cytochrome C. High energy electrons are passed from complexes I and II to coenzyme Q, where they are moved to complex III, cytochrome C, complex IV and their ultimate low energy electron acceptor, oxygen. The energy gained from electron passage along the ETC allows complexes I, III, and IV to pump protons across the inner mitochondrial membrane, generating the mitochondrial membrane potential. The movement of proteins down the electrochemical gradient across the mitochondrial inner membrane to the matrix is coupled to ATP generation by complex V (i.e., ATP synthase) [22]. Importantly, this pathway is integrated closely with other major cellular metabolic pathways. Notably, ETC complex II includes succinate dehydrogenase, a major enzyme in the tricarboxylic acid (TCA) cycle. The TCA cycle is central to cellular energy metabolism; it connects the ETC, glycolysis, the pentose phosphate pathway, amino acid metabolism, and lipid metabolism, which serve as the major metabolic pathways in cells [23]. Ultimately, changes in expression, activity, or function of proteins critical to OXPHOS often have major cellular and systemic pathological effects.

## Mitochondrial dysregulation and disease

The unique features of mitochondria that make them critical to cellular metabolism also make them susceptible to damage, which can contribute to many disease processes. As previously described, mitochondrial heteroplasmy occurs due to the vulnerable nature of mitochondrial DNA and its proximity to a major source of ROS. This heteroplasmy accumulates with normal aging but in excess drives metabolic dysregulation and potentially disease [13, 17, 24]. Changes to nuclear-encoded mitochondrial regulators are also associated with aging and disease; for example, PGC1α, a regulator of mitochondrial biogenesis, is altered in diseases of aging [25, 26]. Moreover, redox imbalance is characteristic of aging-associated mitochondrial dysfunction and OXPHOS defects; impaired antioxidant responses contribute to the inability to respond to damaging ROS, which are often increased, and maintain redox homeostasis [27–30]. In addition to the aforementioned dysfunctional states, mitochondria may exhibit changes to fission and fusion, Ca^2+^ ion signaling, protein and metabolite import, size and structure, as well as other specific perturbations generalized to “mitochondrial dysfunction”. While mitochondrial function and structure have been extensively reviewed [2, 22, 31], the focus of this review is on existing literature examining mitochondrial dysfunction in the context of urologic disease. Given the potential of mitochondrial modulators as a class of pharmaceuticals (e.g, Mito-Q, Quercetin, etc.) [32] understanding the role of mitochondrial dysfunction in disease has substantial potential for improving patient outcomes long-term.

## Mitochondrial diseases and lower urinary tract symptoms

The first description of a mitochondrial-originating disease occurred in 1962. Luft et al. described a hypermetabolic patient of “unknown origin” and upon a muscle biopsy, determined that mitochondrial abnormalities were likely driving the metabolic phenotype [33]. Over the next two decades, the characterization and understanding of mitochondrial-driven diseases expanded extensively. Over 500 mutations have been annotated in both coding and noncoding regions of mtDNA. 130 of those annotated mutations are in protein coding or tRNA loci that are confirmed pathogenic mutations (Cfrm), per the standards used by the MitoMap database [34]. For the purpose of this review, mitochondrial diseases are defined as conditions that arise directly from mutations to mtDNA or nuclear-encoded mitochondrial proteins. Many mitochondrial DNA mutations lead to syndromes with overlapping phenotypes, such as neurological or muscular defects. Given both neurons and muscle cells have high energy demands, they are thought to be more susceptible to mitochondrial defects. Disease phenotypes may also be affected by mutation penetrance, pathogenicity, heteroplasmic frequency, and tissue specificity. Many mitochondrial diseases arising from mtDNA mutations are well defined, but their connection to LUTS is underreported and understudied.

Mitochondrial myopathy, encephalopathy, lactic acidosis, and stroke-like episodes (MELAS) is one of the most frequent mitochondrial genetic disorders. Although associated with many mtDNA mutations, the most common is the m.3243A>G on the MT-TL1 gene, encoding one of two mitochondrial tRNA Leu (UUR) [34]. Given this mutation occurs in the wobble position, it specifically affects ribosomal recognition of the codon, thereby causing impaired translation and synthesis of ETC polypeptides. This leads to the multi-organ dysfunction often present in MELAS patients [35]. While not a direct investigation of LUTS, a study investigating autonomic symptoms in symptomatic carriers of the m.3243A>G mutation revealed a significant increase in bowel or bladder incontinence when compared to non-familial controls [36]. Another study examined adult patients with genetically confirmed mitochondrial disease originating from multiple mutations, both nuclear and mitochondrial in origin, for LUTS. Mitochondrial disease severity was defined by the Newcastle Mitochondrial Disease Adult Scale (NMDAS), and a direct correlation was observed between NMDAS and LUTS severity (Spearman’s ρ  =  0.26, *P*  =  0.011) [37]. One investigation examined adult patients (mean age 46.2 years) with confirmed mitochondrial disease (*n* = 39 females, *n* = 19 males) and controls (*n* = 9 females, *n* = 10 males, mean age 44.0) for incidence of LUTS. The Urinary Symptom Profile (USP) detected LUTS in 83.7% of the combined case population compared to 68.8% controls [38]. When separated by sex, females had more overactive bladder symptoms than their control counterparts; no significant distinctions were observed in case versus control males [38]. Pediatric patients with mitochondrial disease may also present with LUTS. In one study of 15 pediatric patients reporting urinary symptoms, 73% reported urinary retention, and urodynamics revealed a 172% elevated median bladder capacity compared to expected capacity calculated by age [39]. LUTS are not commonly included in the diagnostic criteria for mitochondrial diseases, but their prevalence is high in this patient population.

## Metabolic disease and lower urinary tract symptoms

### Metabolic syndrome

Metabolic syndrome (MetS) is defined by a group of conditions including hypertension, elevated blood glucose and cholesterol, as well as increased visceral fat. MetS raises the risk of heart disease, type 2 diabetes (T2DM), stroke, and obesity [40]. While the mechanisms underlying the development of MetS are unclear, inflammation is thought to contribute to this condition. The impact of mitochondrial dysfunction and oxidative stress on MetS and underlying inflammation has been covered thoroughly in other reviews [41–43].

A lesser-known comorbidity of MetS is LUTS. All four characteristic pathologies of MetS are risk factors for LUTS [44]. For example, hypertension increases the prevalence and severity of LUTS in men [45–47] and both increased blood glucose and cholesterol are positively associated with LUTS [48]. Multiple studies indicate that obesity, waist circumference, and visceral fat are associated with an increased risk of LUTS [49–51]. Much of the investigations examining MetS and LUTS have focused on LUTS resulting from benign prostatic hyperplasia (BPH) [44–46, 48]. Inflammation and oxidative stress have been proposed as mechanisms underlying both MetS and BPH [52]. Mouse models of MetS have recapitulated this phenomenon. For example, while on a high fat diet, the SAMP6 and AKR/J mouse strains both had increased urinary retention and increased void counts compared to low fat diet fed mice [53]. While MetS is a complex, multifaceted syndrome with no clearly defined unifying mechanism, the contribution of mitochondrial dysfunction has been proposed as a driver of the characteristic inflammation and oxidative stress [41]. While a causal mechanism between mitochondrial dysfunction, MetS, and LUTS has not been defined, the correlative data is supportive of further investigation. Moreover, given both LUTS and MetS are more common in older adults, a treatment targeting the underlying mechanism of both syndromes would be particularly advantageous for the aging community, who are otherwise prone to polypharmacy.

### Diabetes

While the mechanisms underlying the development of both type one (T1DM) and T2DM are distinct, they have some overlapping pathophysiology. Of note, inflammation, mitochondrial dysfunction, and oxidative stress are thought to contribute to both conditions [54]. Glucose homeostasis is maintained largely by pancreatic islet cells, which are dependent on OXPHOS for energy [55, 56]. Islet cells respond to changing ATP/ADP ratios for glucose-stimulated insulin release, and impairments to mitochondria can significantly affect this process [57]. Recent literature reveals islets from patients with T2DM have a 90% reduction in PGC1α expression, a master indirect regulator of mitochondrial biogenesis [58]. Furthermore, the activities of mitochondrial enzymes glycerol phosphate dehydrogenase, pyruvate carboxylase, and succinyl-CoA:3-ketoacid-CoA transferase are decreased by 73%, 65% and 92%, respectively, in pancreatic islets compared to controls [59]. The disruption of mitochondrial function in islet cells may play a central role in the impaired insulin secretion of diabetic pancreatic islets.

In addition to mitochondrial manifestations in the pancreas, skeletal muscle is highly impacted by oxidative stress and mitochondrial dysfunction in T2DM. PGC1α responsive nuclear-encoded genes involved in OXPHOS are downregulated in skeletal muscle of patients with T2DM [60, 61]. Mitochondria from the skeletal muscle of diabetic individuals have decreased complex I oxidoreductase activity and citrate synthase activity. Additionally, mitochondrial size is significantly reduced in T2DM patients [62]. Collectively, these studies demonstrate the importance of mitochondrial dysfunction in the pathology of T1DM and T2DM.

Diabetic patients have numerous systemic comorbidities associated with the disease, with detrusor overactivity as the earliest manifestation, and diabetic bladder dysfunction (DBD) as the most common. Occurring in more than 50% of patients [63], DBD includes a wide variety of clinical presentations including decreased bladder sensation, poor contractility, increased post-void residual urine, urge incontinence, and overactive bladder [64]. DBD occurs in patients with both T2DM and T1DM. Data from two large studies revealed an association between T2DM and prevalent LUTS in men [65]. In addition, the National Health and Nutrition Examination Survey revealed an association between diabetes and LUTS in men over the age of 40 [66]. The overall prevalence of any LUTS in the population with a diabetes diagnosis was 52.7% compared with 36.5% in the entire study population (*P* < 0.0001); nocturia, incomplete emptying, and hesitancy were also significantly higher than the population without diabetes [66]. Specifically, the younger age groups (40–69) reported higher incidence of LUTS in diabetes patients than those without. Furthermore, men under the age of 70 with diabetes for longer than five years were more likely to report LUTS than those with shorter disease duration (<5 years) [66]. Length and onset of diabetes may affect onset and severity of LUTS in men over 40.

Studies of T1DM also show an increase in urological complications. Specifically, 65% of women, and 68% of men reported at least one urological complication in an ancillary longitudinal study of T1DM patients [67]. Mouse models also reveal this connection. The nonobese diabetic (NOD) mouse, used as a model of T1DM due to its autoimmune-driven phenotype, also demonstrates a urinary phenotype [68]. In fact, NOD mice can develop spontaneous prostatitis, an inflammatory process thought to contribute to BPH development in some men [69]. Another recent study investigating the effects of hyperglycemia and chronic inflammation on the prostate revealed that NOD mice have increased prostate epithelial proliferation and immune cell infiltration associated with a more severe phenotype [70]. Rat models also demonstrate the importance of mitochondrial function to bladder function in diabetes. One group used the streptozotocin model of induced diabetes and found that an antioxidant dye (IR-61) was taken up by bladder smooth muscle cells, reduced ROS, induced the antioxidant response, and prevented structural damage all while alleviating bladder dysfunction [71]. Current studies on mitochondrial dysfunction in DBD are limited to correlative patient studies and tissue-specific evidence in animal models.

## Prostate dysfunction and mitochondrial dysfunction

Significant progress has been made recently to evaluate the role of mitochondrial dysfunction in BPH, which is a predominant driver of LUTS in aging men. BPH is characterized by some combination of hyperplasia, smooth muscle dysfunction, and/or fibrosis which many times leads to urinary symptoms [72]. Key mitochondrial proteins are decreased in patients with BPH, such as NADH:ubiquinone oxidoreductase core subunit S3 (NDFUS3), which could lead to impaired OXPHOS and broader mitochondrial dysfucntion [73]. This change is recapitulated in the aged mouse model of lower urinary tract dysfunction (LUTD), which also has decreased NDUFS3 expression. Furthermore, prostate cells with induced complex I dysfunction exhibit a pro-fibrotic phenotype [73]. Interestingly, further research has shown that use of common non-steroidal anti-inflammatory drugs (NSAIDs) and finasteride, a first-line BPH treatment, decreases NDUFS3 expression in the prostate of patients with BPH [74]. Mice treated with NSAIDs (celecoxib), also had decreased expression of NDUFS3 [74]. This critical finding suggests that current treatments may paradoxically trigger or exacerbate underlying mitochondrial dysfunction and even contribute to patient treatment failure. Oxidative stress is another potential marker of mitochondrial dysfunction in BPH. Human BPH tissues have higher levels of 8-hydroxy-2′-deoxyguanosine (8-OHdG) and 8-OHdG levels had a weak (R^2^ = 0.35), but significant (*p* = 0.03), correlation with prostate weight [75]. Oxidative stress was also present in a steroid hormone-induced mouse model of LUTD, where proteomics analysis identified changes in proteins associated with the oxidative stress defense and inflammatory response pathways in urine [76]. In addition, prostate tissues from this model had altered levels of multiple proteins involved in the oxidative stress response and H_2_O_2_ metabolism [76].

## Bladder dysfunction and mitochondrial dysfunction

Emerging evidence suggests that mitochondrial dysfunction plays a role in the development and progression of bladder dysfunction. Bladder dysfunction includes a range of LUTS, which can result from a variety of causes. BPH is a common driver of bladder dysfunction, as patients with partial outlet obstruction may decompensate (e.g., bladder wall hypertrophy, impaired detrusor activity). One study demonstrated the mitochondrial enzymes malate dehydrogenase and citrate synthase were significantly decreased in bladder biopsies of men with urologic dysfunction, highlighting the importance of biochemical pathways (i.e., TCA cycle) linked to mitochondrial bioenergetics [77]. Data collected on 52 men with and without bladder outlet obstruction revealed that mitochondrial damage and mean mitochondrial volume correlate with the severity of partial outlet obstruction [78]. The authors report evidence of ischemic injury with worsening outlet obstruction, and note that other fields have correlated ischemic injury with worsening mitochondrial structural damage [78].

In addition to human studies, animal models of bladder dysfunction show mitochondrial dysfunction. In a study investigating rabbit bladders after partial outlet obstruction, increased tissue hypertrophy and decreased bladder contractility correlate closely with alterations of mtDNA content and mitochondrial-related nuclear genomic alterations in smooth muscle [79]. Perhaps their most interesting finding was that mtDNA content correlated with the capacity of bladder function to return to normal after alleviation of obstruction [79]. Thus, while mitochondrial dysfunction may be secondary to the initiation of outlet obstruction, it may become a regulatory factor in healing once the obstruction is alleviated. Another study in mice demonstrated the importance of mitochondria to the healthy bladder; *Pparg* was shown to regulate mitochondrial biogenesis and fatty acid transport in urothelial cells and regulates the differentiation and/or survival of multiple cell subtypes [80]. Aged rats (25–30 months) also demonstrated changes to the mitochondria of the urothelium; cytochrome C and reactive oxygen species were increased in aged bladders compared to young (3–4 months). Cultured isolated urothelial cells from aged rats also had lower mitochondrial membrane potential and decreased basal respiration and ATP production [81]. Further studies in humans are needed to validate the clinical relevance of these findings.

## Incontinence and mitochondrial dysfunction

Stress urinary incontinence (SUI) is a common condition among women which increases with age. While SUI is often attributed to weakening of the pelvic floor and rhabdosphincter, mitochondrial function contributes to these processes. Transmission electron microscopy of the rhabdosphincter of older patients revealed signs of morphological abnormalities, including mitochondrial swelling, increased mitochondrial area, and vacuolation [82]. Moreover, oxidative stress is present in the fibrous connective tissue of the pelvic floor, which is thought to further cause mitochondrial homeostatic dysregulation [83]. Specifically, patients with SUI had increased levels of the oxidative damage markers 8-OHdG (DNA damage) and 4-HNE (lipid damage) in anterior vaginal wall tissue [83]. Furthermore, the same study showed decreased mtDNA content, suggesting decreased mitochondrial number, decreased mitophagy, and decreased regulators of mitochondrial biogenesis [83]. A common cause of SUI, pelvic organ prolapse, has also been studied in connection with mitochondrial dysfunction and found similar findings including oxidative stress, decreased mtDNA content, and increased mitochondrial mutations [84, 85]. As with other urinary conditions, targeting mitochondrial dysfunction may open new treatment avenues for SUI. Furthermore, while incontinence is a less common LUTS experienced by men, it does occur in association with some surgeries performed for prostate-driven symptoms [86], and the role of mitochondrial dysfunction remains unstudied.

## Conclusion

Mitochondria are unique and complex organelles, deriving from their origin as individual microbiota. Their genome is more susceptible to damage than nuclear DNA yet both genomes encode proteins that are essential for the bioenergetic functions of mitochondria. Given the complexity and necessity of mitochondria, defects in the structure or function of these organelles are often pathogenic. Specifically, many genetic defects occur within components of the ETC, which is central to many mitochondrial functions including but not limited to ATP production, mitochondrial membrane polarization, redox environment balance, ROS signaling, and metabolic homeostasis. Disruptions in any mitochondrial function can contribute to common processes such as inflammation, fibrosis, proliferation, and oxidative stress, and accumulate during aging. Current gaps in knowledge exist in many areas of study. There is substantial correlative evidence, reviewed herein (Table 1), that mitochondrial dysfunction is involved in LUTS—both in diseases that affect the urogenital tract directly (e.g., bladder dysfunction, BPH) and those causing LUTS indirectly (e.g., genetic mitochondrial disease, diabetes). Aging is an underlying risk factor of many of these conditions and may contribute to mitochondrial dysfunction, which is a known hallmark of aging. Furthermore, there remains the question of whether mitochondrial dysfunction is an inducer of disease or a byproduct of other cellular dysfunction. Regardless of the origin of mitochondrial dysfunction, the potential use of pharmaceuticals and supplements with mitochondrial modulating properties is of great interest, especially since they may be able to target shared pathology, improving overall health and avoiding polypharmacy. Current studies provide an excellent justification for further investigation into mitochondrial dysfunction as a driver of non-malignant urologic disease.

**Table 1:** Summary of the relevant mitochondrial phenotypes, urinary phenotypes, specificity of the mitochondrial phenotype, and corresponding references for the conditions reviewed herein.

Disease	Mitochondrial phenotype	Urinary phenotype	Direct vs indirect phenotype	References
Inherited Genetic Disease	mtDNA mutation	LUTS, incontinence, urinary retention	Indirect	[36–39]
Metabolic Syndrome	Oxidative stress	LUTS, BPH	Indirect	[41–53]
Diabetes	Oxidative stress, decreased mitochondrial protein expression	DBD, LUTS	Indirect	[54–71]
Benign Prostatic Hyperplasia (BPH)	Oxidative stress, decreased mitochondrial protein expression, ROS, increased 8-OHdG	LUTS, urinary retention	Direct (prostate)	[72–77]
Bladder Dysfunction	Decreased TCA proteins, mitochondrial structural damage, decreased mtDNA content, increased ROS, decreased respiration	LUTS, urinary retention, decreased bladder contractility, bladder wall hypertrophy	Direct (bladder)	[78–81]
Stress urinary incontinence (SUI)	Mitochondrial structural damage, oxidative stress, increased 8-OHdG and 4-HNE, decreased mtDNA content, mtDNA mutations, decreased mitophagy	Incontinence, weakening pelvic floor	Direct (pelvic muscles)	[82–85]
